# Supplementary material for: Olaparib Conjugates with Selenopheno[3,2-c]quinolinone Inhibit PARP1 and Reverse ABCB1-Related Multidrug Resistance
Source: Pharmaceutics. 2022 Nov 23;14(12):2571. doi: 10.3390/pharmaceutics14122571 (PMC9783898; doi:10.3390/pharmaceutics14122571)
Supplement: Supplementary file 1 [file pharmaceutics-14-02571-s001.zip › pharmaceutics-1976487-supplementary.pdf]

Olaparib conjugates with selenopheno[3,2-*c*]quinolinone inhibit PARP1 and reverse ABCB1-related multidrug resistance

Marina Makrecka-Kuka, Jelena Vasiljeva, Pavels Dimitrijevs, Pavel Arsenyan*

File S1: The copies of 1H, 13C, 19F, 77Se NMR, HRMS, and HPLC

^{1}H NMR of **5a**

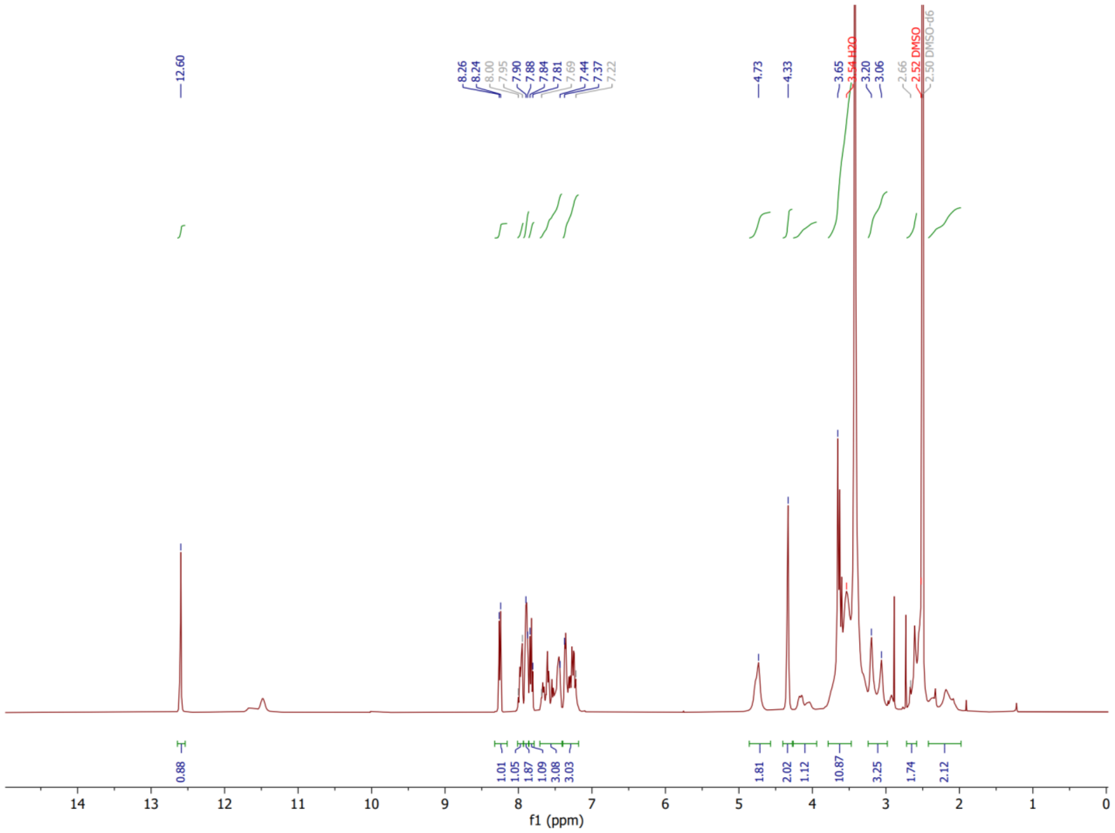

^{13}C NMR of **5a**

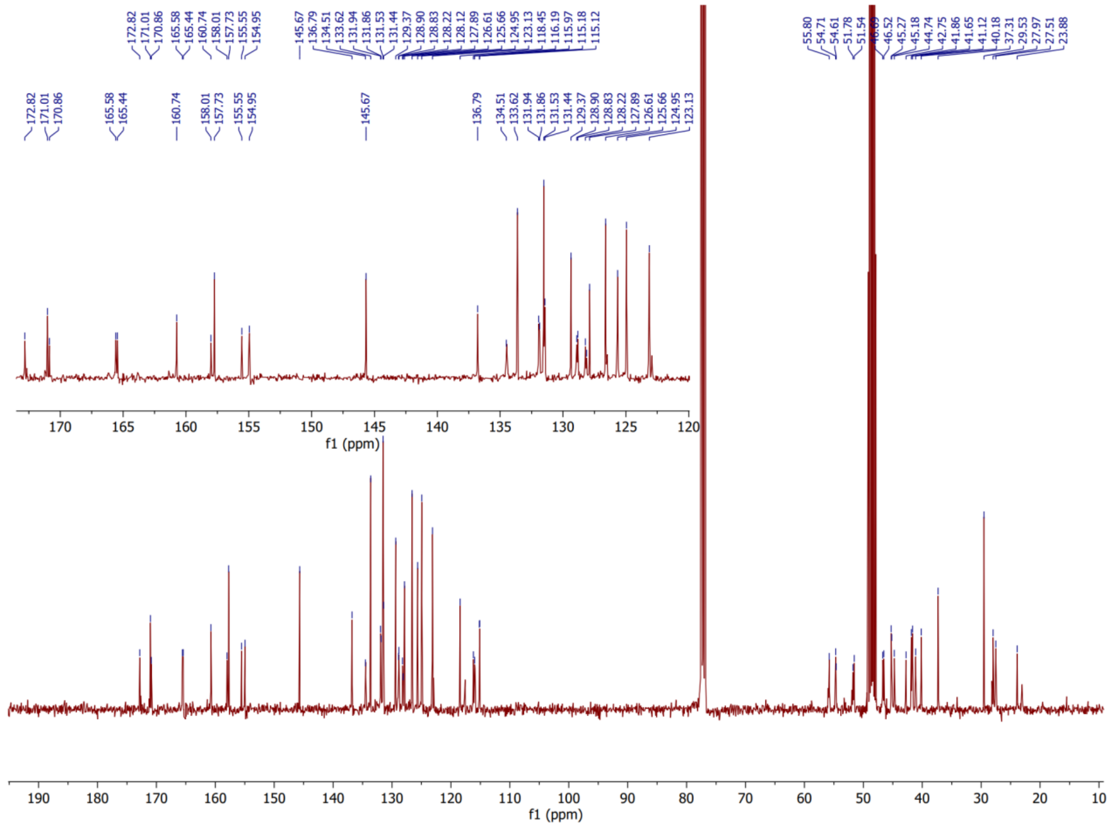

^{19}F NMR of **5a**

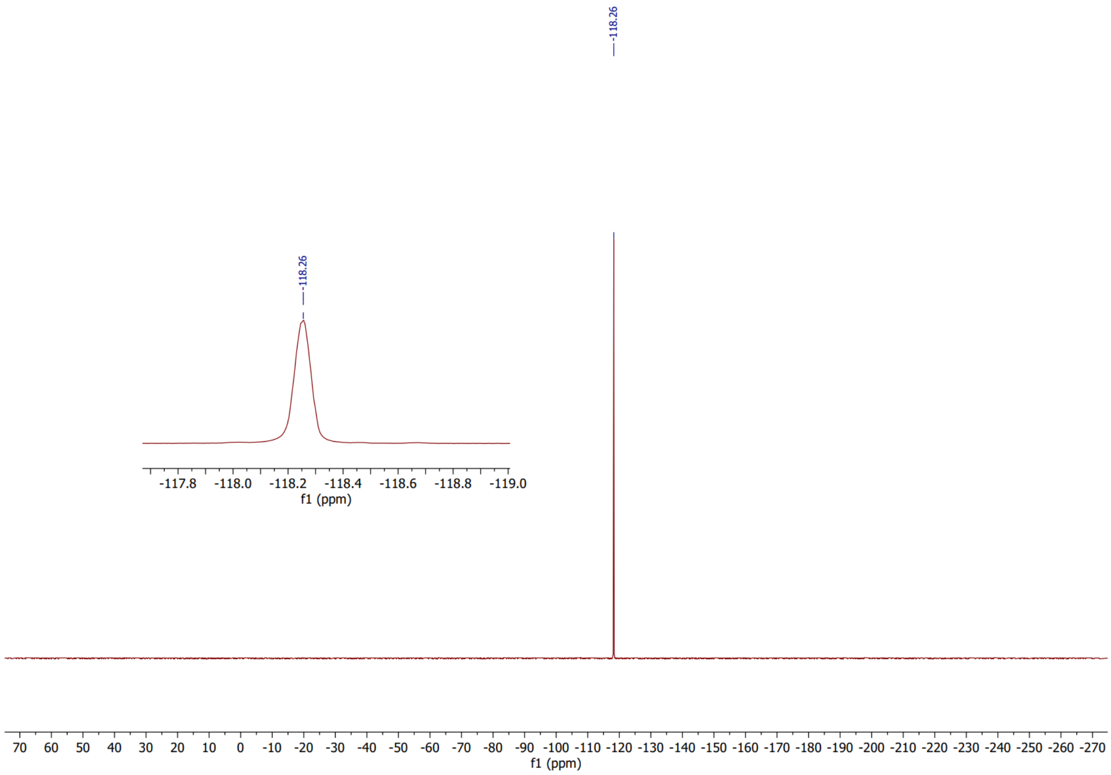

^{77}Se NMR of **5a**

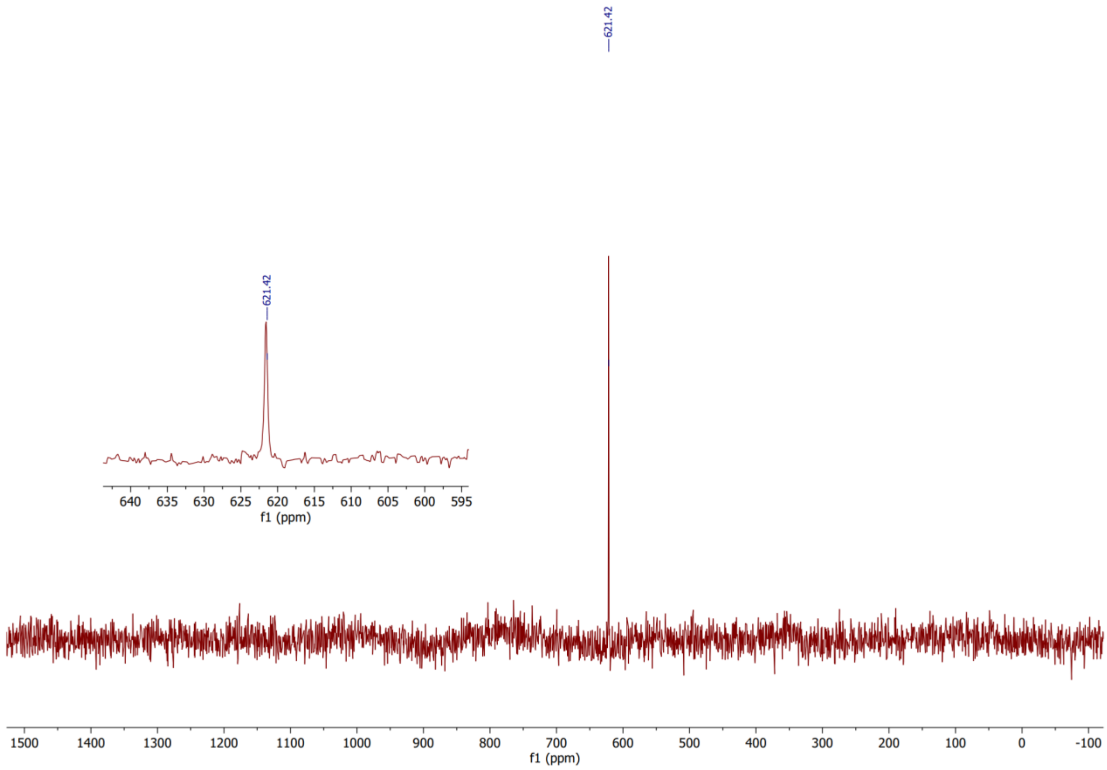

HRMS of **5a**

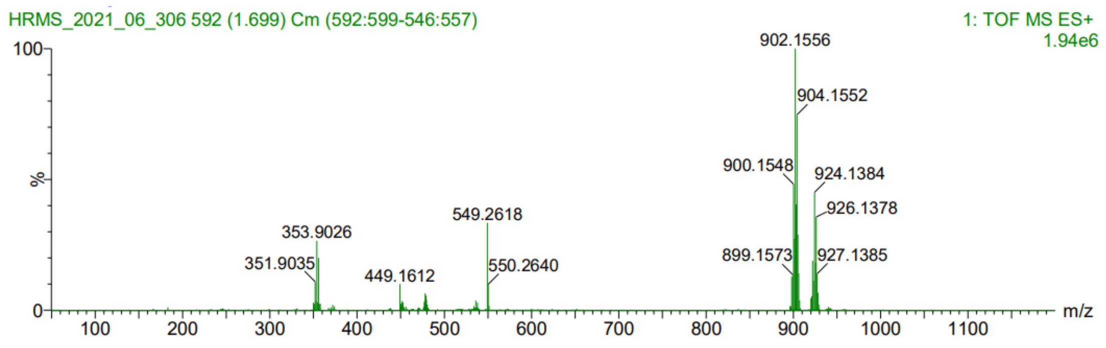

HPLC of **5a**

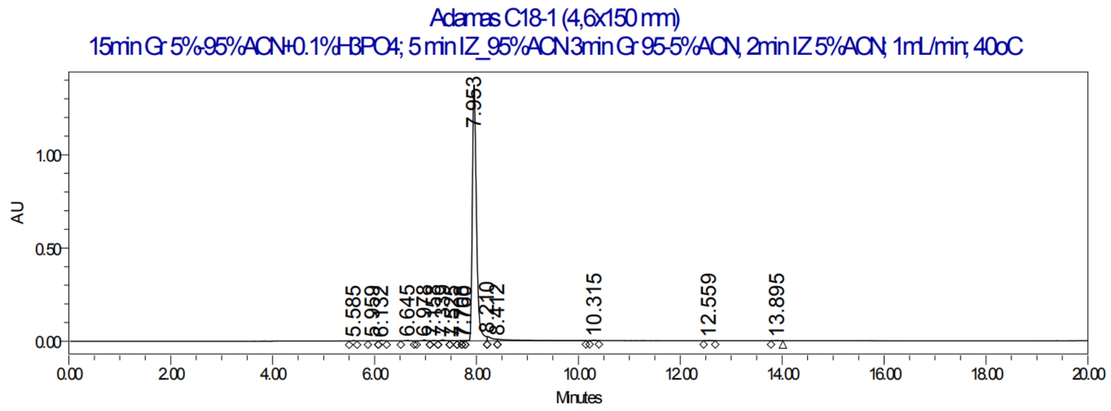

	RT	Area	%Area	Height	EP Plate Count	Width@50%	Resolution	Selectivity
1	5.585	7645	0.09	2263	72675	0.0488		
2	5.959	5206	0.06	637	13352	0.1214	2.592	1.092
3	6.132	8815	0.11	2254	70801	0.0542	1.161	1.039
4	6.645	16074	0.19	3017	41615	0.0767	4.626	1.111
5	6.978	23133	0.28	4624	63808	0.0650	2.773	1.065
6	7.158	11150	0.13	2062	39273	0.0850	1.420	1.033
7	7.339	28302	0.34	5024	62254	0.0692	1.381	1.032
8	7.525	7007	0.08	1065				1.032
9	7.708	5001	0.06	1127				1.030
10	7.760	5090	0.06	1440				1.008
11	7.953	7742595	93.66	1372844	51512	0.0825		1.031
12	8.210	166811	2.02	22060				1.040
13	8.412	217775	2.63	7869				1.030
14	10.315	12332	0.15	2412	94327	0.0790		1.275
15	12.559	5029	0.06	766	63722	0.1171	13.500	1.255
16	13.895	5168	0.06	929	152563	0.0837	7.850	1.121
Sum		8267131.9						

^{1}H NMR of **5b**

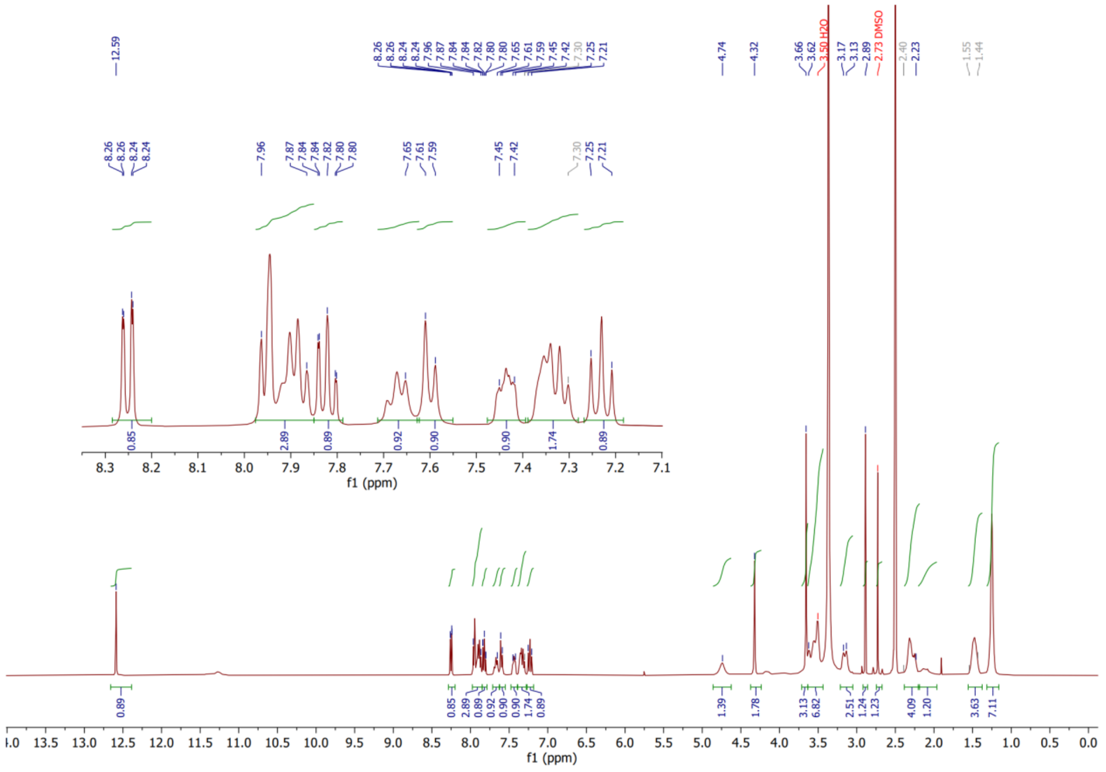

^{13}C NMR of **5b**

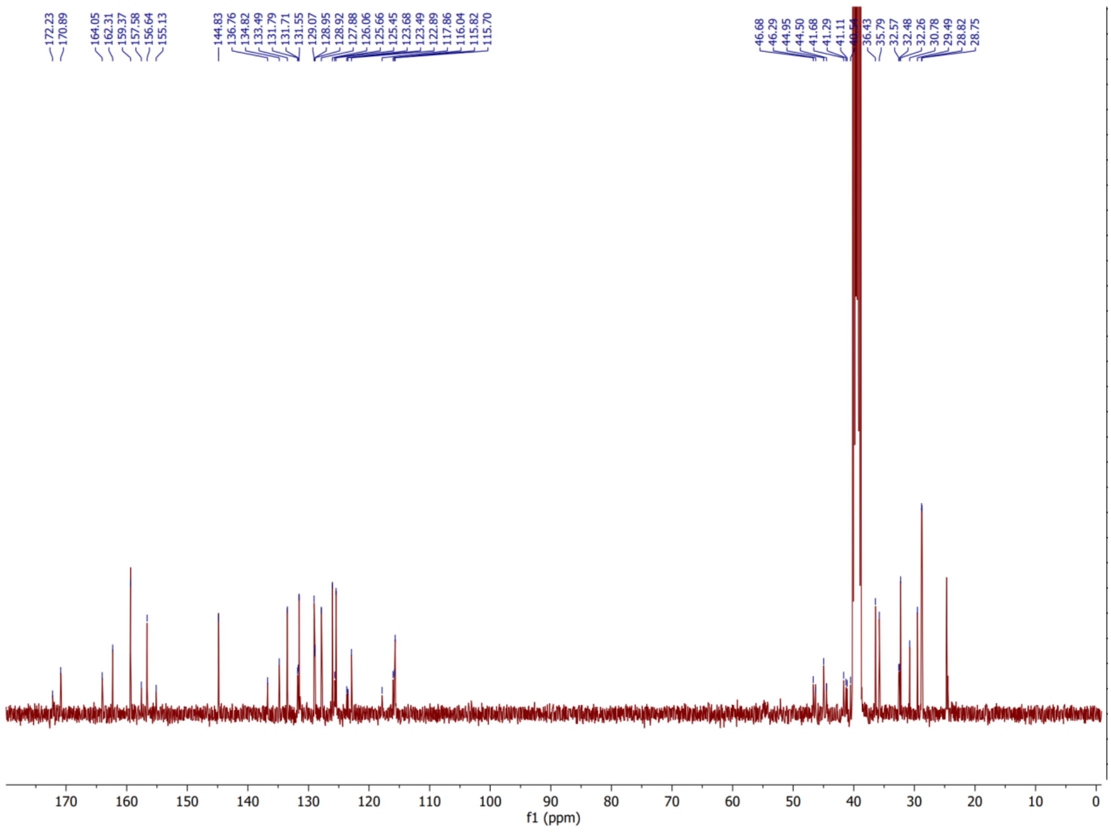

^{19}F NMR of **5b**

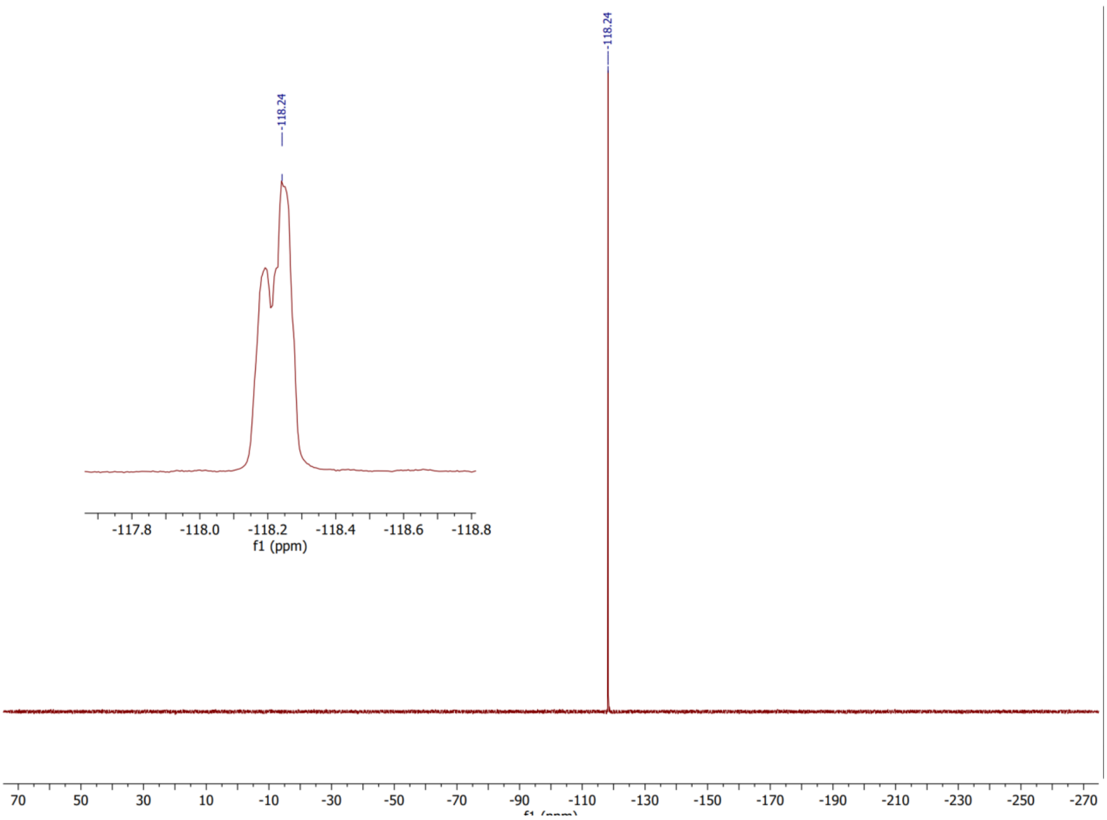

^{77}Se NMR of **5b**

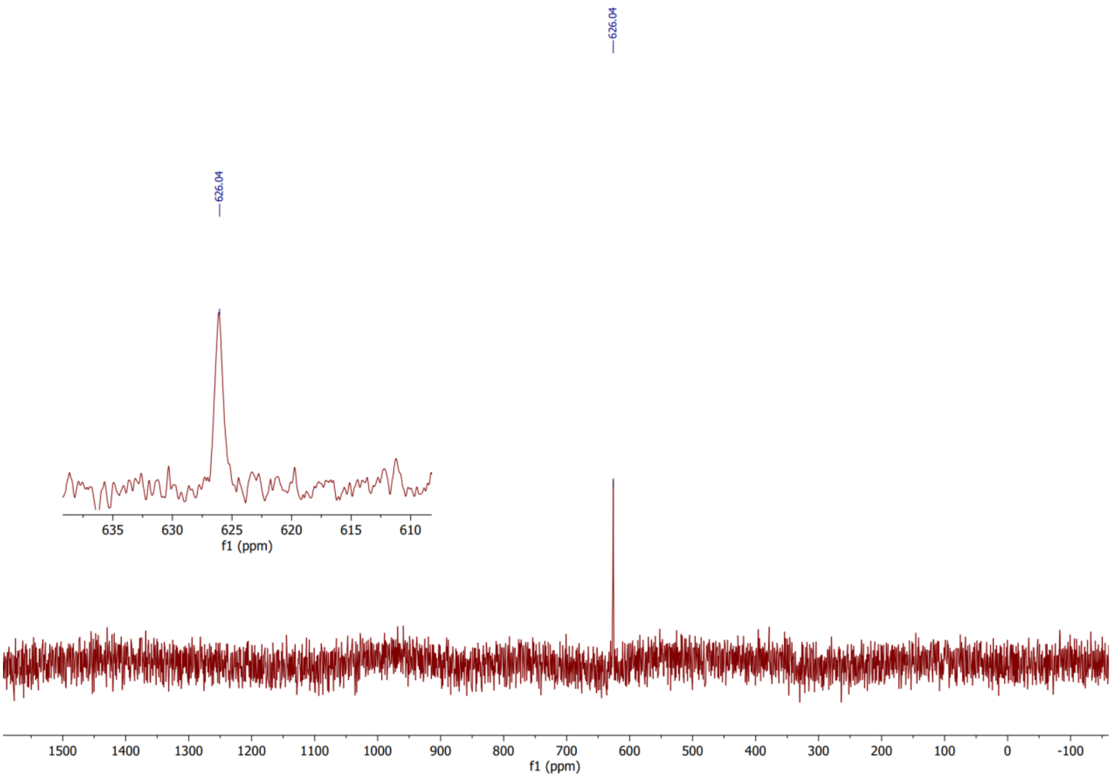

HRMS of **5b**

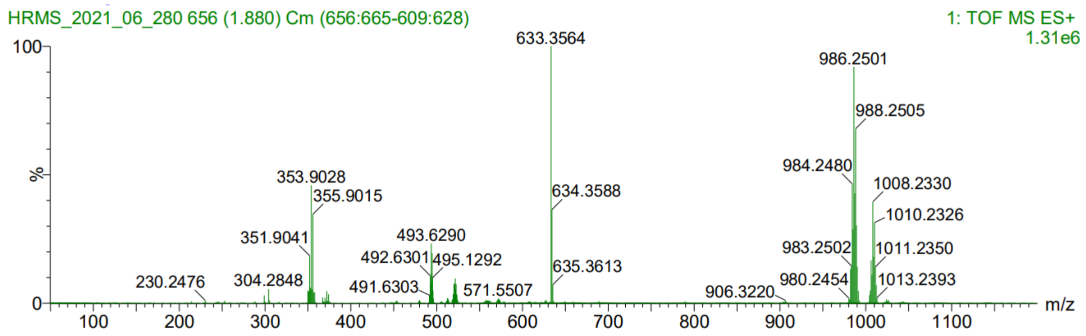

HPLC of **5b**

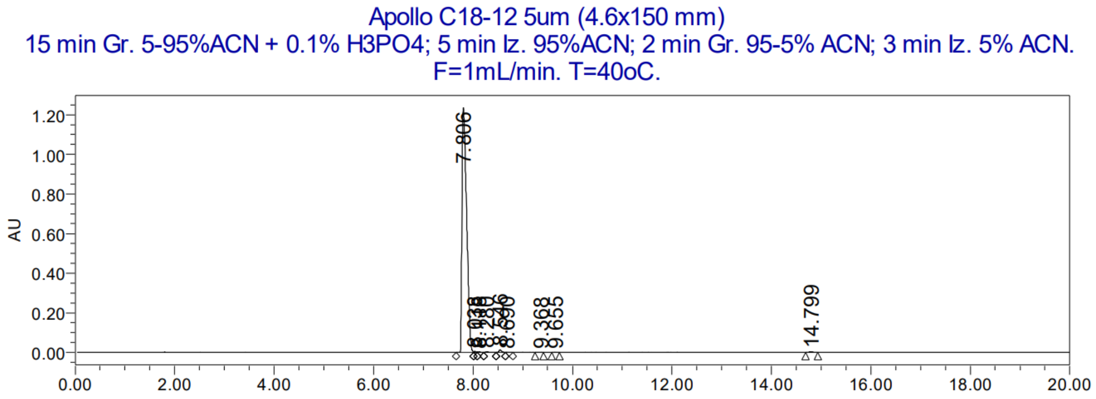

	RT	Area	% Area	Height	EP Plate Count	Resolution	Selectivity	Width @ 50%
1	7.806	8022125	98.61	1235329	30257			0.106
2	8.038	15509	0.19	4798			1.04	
3	8.118	8373	0.10	1868			1.01	
4	8.280	15355	0.19	3527	147473		1.02	0.051
5	8.546	49465	0.61	13692	146210	3.04	1.04	0.053
6	8.690	3991	0.05	1013	114337	1.50	1.02	0.060
7	9.368	2188	0.03	440	76663	5.71	1.10	0.080
8	9.655	2673	0.03	793	192627	2.58	1.04	0.052
9	14.799	15632	0.19	3761	288834	52.06	1.64	0.065

^{1}H NMR of **5c**

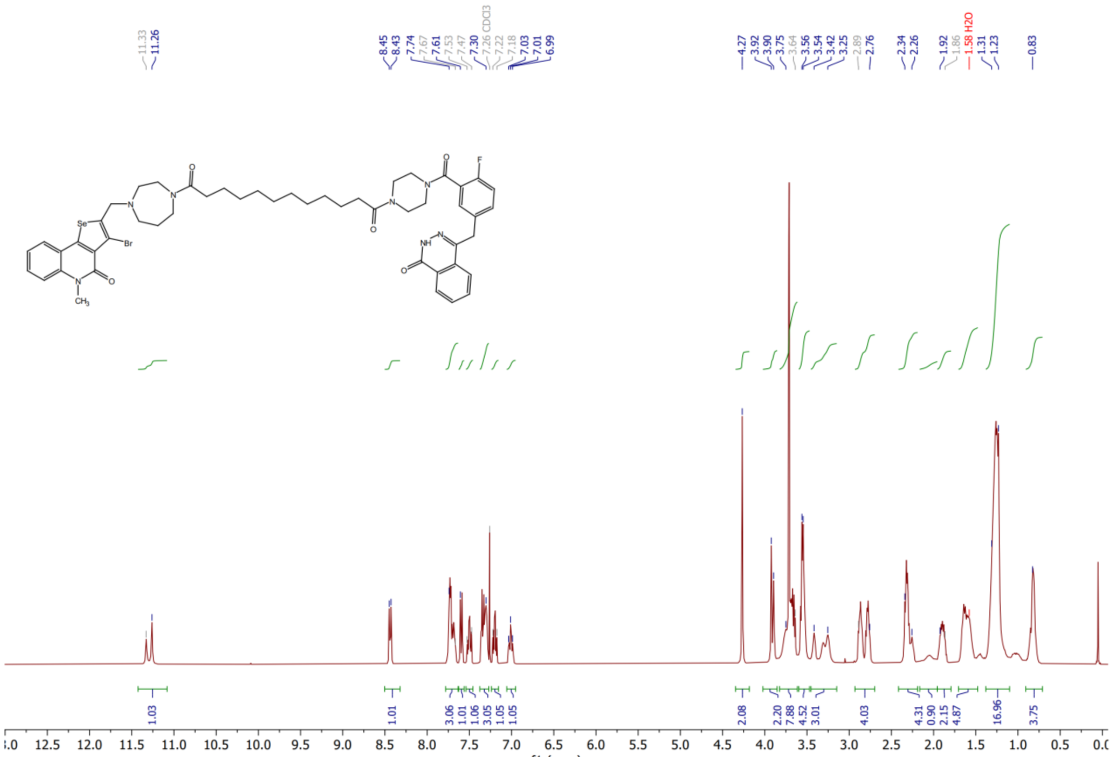

^{13}C NMR of **5c**

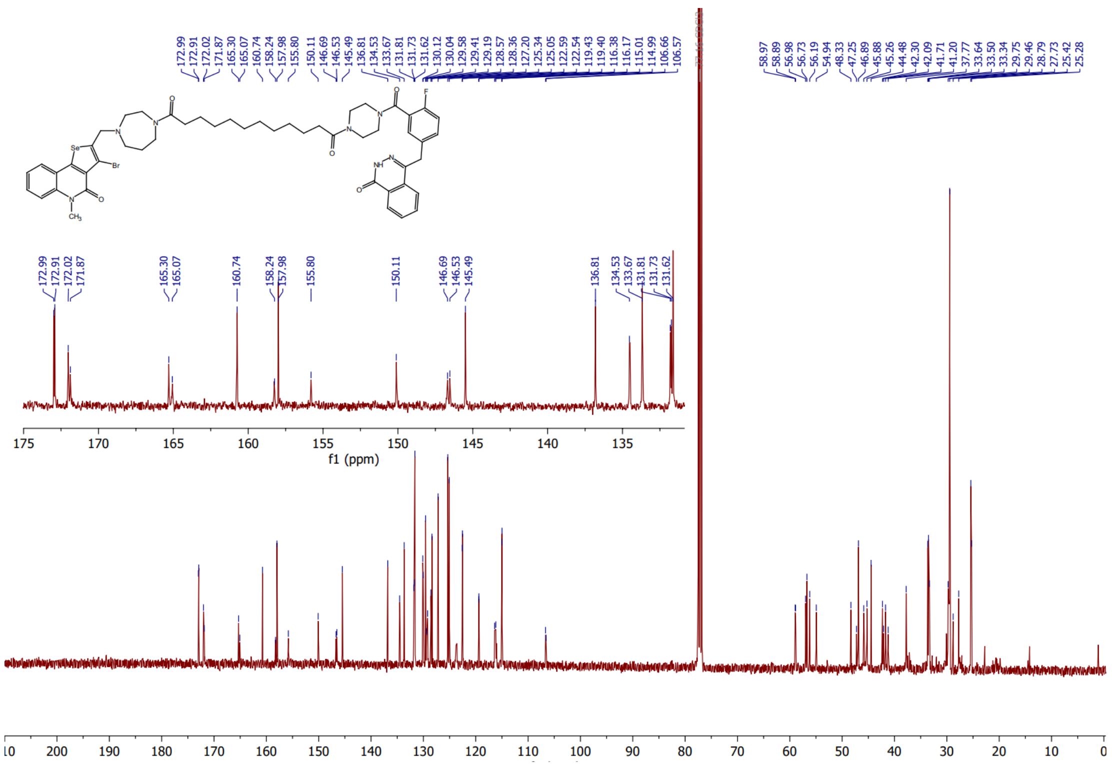

^{19}F NMR of **5c**

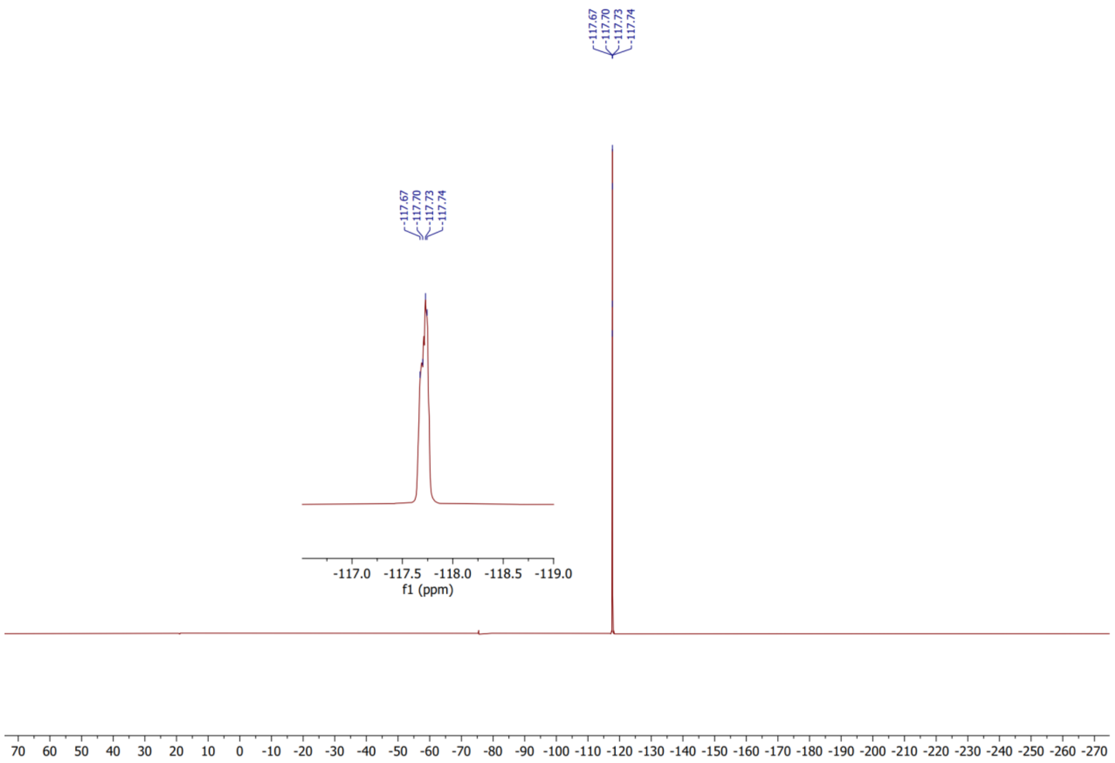

^{77}Se NMR of **5c**

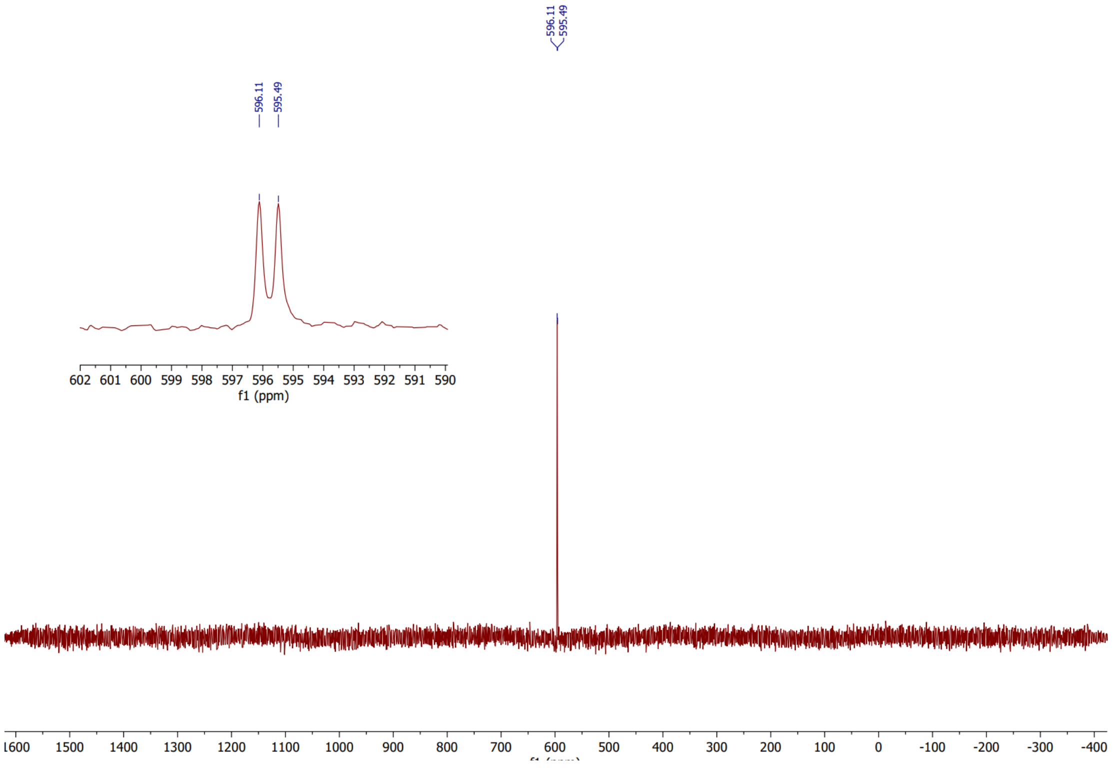

HRMS of **5c**

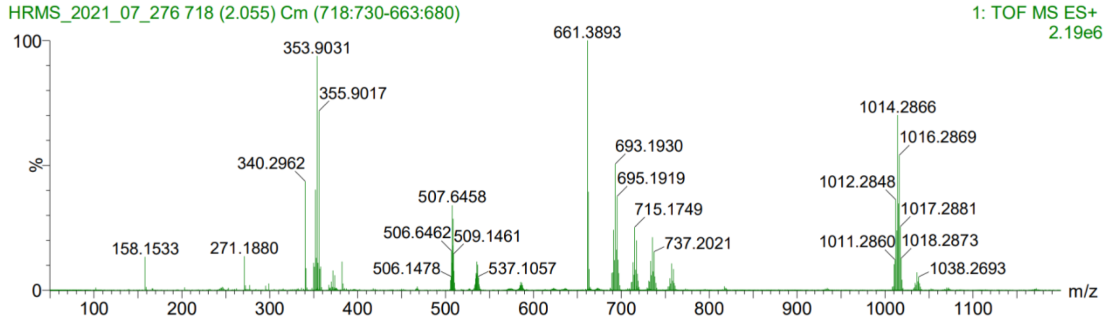

HPLC of **5c**

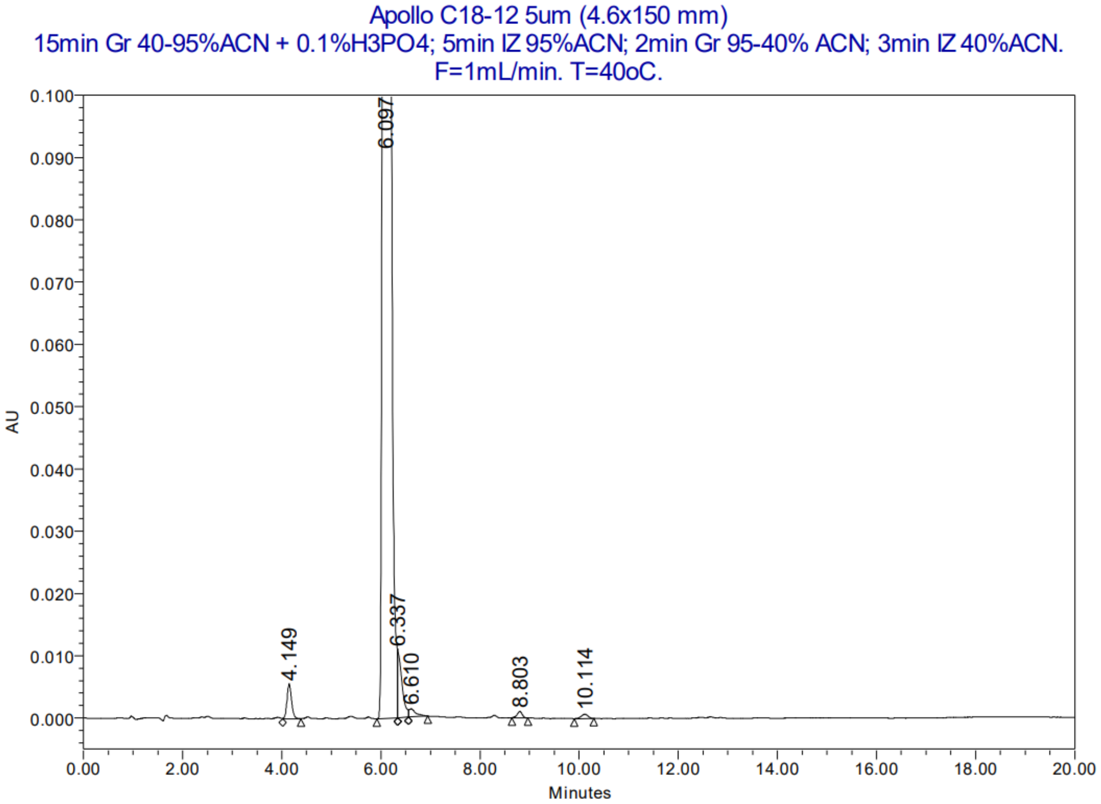

C=0,45mg/ml (40% ACN+0.1%PA)

	RT	Area	% Area	Height	EP Plate Count	Resolution	Selectivity	Width @ 50%
1	4.149	35792	0.72	5566	9942			0.098
2	6.097	4830626	97.40	637118	15057	10.70	1.75	0.117
3	6.337	66229	1.34	10952			1.05	
4	6.610	13075	0.26	1298			1.06	
5	8.803	7234	0.15	1041	38333		1.43	0.106
6	10.114	6793	0.14	696	23752	5.95	1.18	0.154
